# Identification of a Hematopoietic Cell Population Emerging From Mouse Bone Marrow With Proliferative Potential *In Vitro* and Immunomodulatory Capacity

**DOI:** 10.3389/fimmu.2021.698070

**Published:** 2021-08-03

**Authors:** Catalina-Iolanda Marinescu, Mihai Bogdan Preda, Carmen Alexandra Neculachi, Evelyn Gabriela Rusu, Sinziana Popescu, Alexandrina Burlacu

**Affiliations:** Laboratory of Stem Cell Biology, Department of Regenerative Medicine, Institute of Cellular Biology and Pathology “Nicolae Simionescu”, Bucharest, Romania

**Keywords:** mesenchymal stromal cells, Ly-6C, bone marrow-derived proliferating hematopoietic cells, CCL-6, immunomodulation

## Abstract

There is continuing interest in therapeutic applications of bone marrow-derived mesenchymal stromal cells (MSC). Unlike human counterparts, mouse MSC are difficult to propagate *in vitro* due to their contamination with adherent hematopoietic cells that overgrow the cultures. Here we investigated the properties of these contaminating cells, referred to as bone marrow-derived proliferating hematopoietic cells (BM-PHC). The results showed that both BM-PHC and MSC had strong immunomodulatory properties on T cells *in vitro*, with PGE2 and NO involved in this mechanism. However, BM-PHC were stronger immunomodulators than MSC, with CCL-6 identified as putative molecule responsible for superior effects. *In vivo* studies showed that, in contrast to BM-PHC, MSC endorsed a more rapid xenograft tumor rejection, thus indicating a particular context in which only MSC therapy would produce positive outcomes. In conclusion, bone marrow contains two cell populations with immunomodulatory properties, which are valuable sources for therapeutic studies in specific disease-relevant contexts.

## Introduction

There has been a continuing interest in the potential therapeutic applications of adult stem-like cells, referred to as mesenchymal stromal cells (MSC). These cells, residing in almost all postnatal organs and tissues, are heterogenous populations of fibroblast-like cells and have initially drawn attention due to their capacity to support hematopoiesis and differentiate into specific cell types (1–4). Within the bone marrow, MSC are known to reside in a complex microenvironment and together with hematopoietic stem cells (HSC) form a unique bone marrow niche (5, 6). HSC produce all blood cell lineages during homeostasis and stress in a highly dynamic program being tightly regulated by an interdependent network with MSC (5).

Among the various types of stem cells proposed for cell therapy (7), MSC were shown to have distinct advantages, which include convenient isolation (MSC can be rapidly obtained from bone marrow and adipose tissue by non-invasive methods), reduced immunogenicity, lack of ethical controversy, and trophic activity (8, 9). Although larger than other stem cells used in cell therapy, MSC can trigger the outcomes with no need of homing to the site of injury, as recent studies pointed towards a remote blood-borne-mediated pathway activated by transplanted MSC (10–13).

In preclinical settings, MSC demonstrated consistent ability to promote tissue healing, modulate inflammation and improve the outcomes in various animal models (14). All these positive *in vivo* effects are primarily due to a broad array of secreted bioactive factors, collectively referred to as MSC secretome, as it is now generally accepted that transplanted MSC do not survive for long *in vivo* (15, 16). The recognition that MSC create a microenvironment suitable for tissue repair has increased the interest in MSC therapy and this interest has been even fueled over the past years by multiple studies showing strong immunomodulatory properties (17, 18) with the principal effector being potent inhibition of T cell function (19, 20). Today, MSC are recognized as promising agents for the treatment of inflammatory disorders due to their immunomodulatory functions in contexts linked to auto/allo-immunity (21, 22).

Several mechanisms of immunomodulation have been proposed for bone marrow-derived MSC isolated from multiple species. Among these, MSC are capable of educating B cells and inducing regulatory B cell production (23). MSC can also polarize the responses of macrophages from a pro-inflammatory to an anti-inflammatory/reparative phenotype (24). They can also function to prevent the maturation of dendritic cells and the lytic ability of natural killer cells (1). Concisely, MSC could have extensive interactions with every major component of the innate and adaptive immune system, through a combination of wide-ranging molecular mechanisms involving paracrine activity, extracellular matrix remodeling, direct contact-based signaling, or extracellular vesicles (25). Identifying the particular molecules contributing to the positive effects in each clinical scenario is important for accelerating the transition into clinical practice, which is still considerably difficult.

Their heterogeneity and the absence of a specific MSC-defining antibody make these cells difficult to characterize. Therefore, mouse MSC are currently defined by using a panel of acceptable surface markers (including Sca-1, CD44, CD105), plastic adherent fibroblast-like growth and functional properties (26, 27). Besides, owing to the limited amount of these cells in the adult mouse, most of our knowledge of the biological properties of MSC has been obtained from the study of *in vitro* expanded MSC, rather than of endogenous (or primary) cells (22). Even so, while human and rat MSC are relatively easily obtained *in vitro*, the expansion of their mouse counterparts is far more difficult. Numerous reports documented that mouse bone marrow-derived MSC are frequently contaminated by hematopoietic progenitors that overgrow the culture during the initial passages. As a result, several strategies to deplete the contaminating cells and purify MSC cultures have been developed by various laboratories (28–32).

In this paper, we comparatively analyzed cells within MSC culture at different passages: an initial passage, at which the hematopoietic cells were prevailing, and two advanced passages, at which MSC culture was free of hematopoietic cells. We showed that both MSC and hematopoietic cells had high immunomodulatory effects on splenic T cells *in vitro*. Analysis of the secretome produced by these cells and inhibitory studies revealed both common and particular molecules involved in their effects. Our data showed that while both cell types had strong immunomodulatory effects on T cells *in vitro*, they were differing in other modulatory properties, such as anti-tumor effect *in vivo*.

## Materials and Methods

### MSC Isolation

MSC were isolated as previously reported (28). Briefly, bone marrow aspirate was obtained from 6-week-old C57Bl/6 mice by flushing the medullar channels of the tibiae and femurs with 5 ml culture medium (DMEM containing 10% MSC-qualified FBS) using a syringe with a 26-gauge needle. A single cell suspension was thereafter obtained by passing the aspirate through increasing needle gauges (from 21 to 25), which was subsequently seeded on 10-cm cell-culture treated Petri dish and incubated at 37°C under 5% CO_2_ atmosphere. The first two passages were performed at around 7-day intervals, by using 0.25% trypsin and gentle scraping with the rubber policeman. Recovered cells were plated at 5000 cells/cm^2^. Starting from the third passage, the cells were trypsinized when reached around 80% confluency, without using the rubber policeman, and replated on 0.1% gelatin-coated plates at 5000 cells/cm^2^.

### Flow-Cytometry

Cells were trypsinized to obtain a single cell suspension and the density was adjusted to 10^6^ cells/ml. One hundred-µl cell suspension was incubated with fluorescent-labeled antibody specific for CD45, Sca-1, CD44, CD29, CD90, CD73, CD105, CD11b, F4/80, CD206, Ly-6C, alone or in combination. All antibodies were purchased from BioLegend. After 30 minutes of incubation at 4°C, the cells were washed by centrifugation and resuspended in FACS buffer (PBS containing 2% fetal bovine serum) for flow cytometry analysis. Propidium iodide (0.2 ug/ml final concentration) was added before analysis to identify the live cells and at least 30,000 events were considered for each sample. Acquired data was analyzed using CytExpert software (Beckman Coulter). For multiple staining, the compensation matrix was obtained using compensation beads (Thermo Fisher Scientific) combined with fluorescent antibodies for every single-color sample.

### Suppression of T-Cell Proliferation by MSC

To assess the ability of MSC to suppress T-cell proliferation, splenic T cells were isolated by nonadherence to nylon (33). T cells were CFSE-labeled and then co-cultured for three days with anti-CD3/CD28 activating microbeads (in a cell: bead ratio of 1:1) in 96-well tissue culture plates at 10^5^ cells per well in the presence or absence of irradiated MSC. Various numbers of MSC (ranging from 625 to 10,000 cells/well) were used to assess their immunosuppression capacity. MSC irradiation was performed 24 hours prior to the interaction with T cells, as previously described (34). The co-culture was maintained for three days, after which the proliferation of the fluorescent cells was analyzed using CFSE dye dilution assay and ModFit software. In experiments assessing various molecules as potential inhibitors for MSC immunosuppressive effect, these molecules were added simultaneously with the lymphocyte suspension at the time of co-culture initiation.

### Cytokine Array

The profiles of the relative levels of cytokines in the conditioned medium produced by MSC culture at low and high passages were analyzed using Proteome Profiler Mouse XL Cytokine Array (R&D Systems). Briefly, the conditioned medium was incubated overnight with the array, followed by a wash step and incubation with a cocktail of biotinylated detection antibodies. Streptavidin-HRP and chemiluminescent detection reagents were then applied, and the signal produced at each spot (corresponding to the amount of protein bound) was detected with FUJIFILM Luminescent Image Analyzer LAS-3000. The pixel densities were analyzed with TotalLab Quant software.

### LEGENDplex Assay

To assess soluble analytes secreted by MSC cultures at different passages, two LegendPLEX mouse panels (Th1/Th2 T Helper Cytokine Panel Version 2 and Mouse HSC Myeloid Panel) were used (BioLegend), according to the manufacturer’s instructions. Briefly, the analytes were measured using bead-based sandwich immunoassays, which captured each soluble analyte between two antibodies. The analytes were bound by specific capture bead populations within a mixture of bead populations, which are differing in size and level of APC fluorescence, and each had specific antibody for a particular analyte on the surface. The concentration of each particular analyte was determined based on a known standard curve using the LEGENDplex™ data analysis software. The following panel of soluble analytes were measured in the supernatant of cells at various passages: IL-5, IL-34, GM-CSF, M-CSF, CXCL12, TGF-β1, SCF, IFNγ, IL-2, IL-4, IL-6, IL-10, IL-13, TNFα.

### ELISA

Concentrations of IL-1ra, CCL-6, HGF, Fractalkine, and Tissue Factor were determined from conditioned medium, using mouse ELISA duo set kits (R&D Systems), following the manufacturer’s protocols. Ang-2 and PGE2 were determined with a Mouse/Rat Angiopoietin-2 Quantikine ELISA Kit and Prostaglandin E2 Parameter Assay Kit, respectively (both from R&D Systems), following the manufacturer’s protocols.

### NO Determination

The ability of the cells to produce NO was assessed by measuring the concentration of nitrite in the culture medium using Griess reagent, according to the manufacturer’s instructions. Briefly, 100 μl conditioned medium was incubated with 50 μl 1% sulfanilamide and 50 μl 0.3% N-1-naphthylethylenediamine dihydrochloride (in 2.5% H_2_SO_4_) for 30 minutes in the dark, to produce a colored azo product. The azo dye product was then spectrophotometrically quantitated based on its absorbance at 548 nm, using a freshly prepared sodium nitrite standard curve.

### xCELLigence Analysis of Macrophage Activation

The effect of MSC on macrophage activation was evaluated with xCELLigence system (Roche Applied Science), using murine macrophage cell line Raw 264.7 and LPS (10 ng/ml) for cell activation. xCELLigence system monitors cellular events in real time by measuring electrical impedance in E-plates, as previously described (9). Cell activation is displayed by increasing cell index in cells treated with LPS. Briefly, 4 x 10^4^ cells were seeded onto each E-plate well in 200 μl DMEM in the presence of LPS and 10% MSC-conditioned medium (CM) which was 10 times concentrated prior to analysis. Concentrated MSC-CM was obtained by ultrafiltration using centrifugal filter units with 3-kDa cut-off (Millipore) and stored in aliquots at -20°C until use. Controls of cells incubated with growth medium (negative control) and LPS-containing medium (positive controls) were also included.

### Xenotransplantation of Tumor Cells

Mice were used in accordance to national and EU regulations for animal experimentation (Directive 2010/63/EU of the European Parliament) and all the procedures were approved by the Ethical Committee of ICBP. Mice were subcutaneously injected into the interscapular region with 50 µl of cell suspension composed of 2x10^6^ U87MG-luc2 cells, alone or mixed with 10^6^ MSC. Tumor development was monitored by *in vivo* imaging system, as described (35). Briefly, mice were intraperitoneally injected with luciferin (150 mg/Kg body weight) and 15 minutes later, they were imaged in dorsal position with IVIS Spectrum system (Perkin Elmer). The following settings were used: field of view 6.6; binning factor 4; F-stop 2; exposure 15 seconds. Surface images were then analyzed using Living Image 4.3.1 software (PerkinElmer, Norway) and quantification of bioluminescence was performed by manually defining regions of interest and reported as photons/second/square centimeter/steradian. Six mice were sacrificed at 5 days after cell injection and cellular pellet was harvested for RNA isolation and Real-time RT-PCR analysis.

### Real-Time RT-PCR Analysis

Total RNA (1 ug) was revers-transcribed into cDNA using High-Capacity RNA-to-cDNA Kit (Applied Biosystems). The qRT-PCR was carried out using SYBR™ Select Master Mix (Applied Biosystems) with 400 nM primer mix at a final reaction volume of 10 µL, on ViiA™ 7 Real-Time PCR System. The cycling conditions were: 50°C for 2 min (UNG activation step), followed by 95°C for 2 min (enzyme activation step), and 40 cycles of amplification (95°C for 1 sec and 60°C for 30 sec). Relative expression was calculated using the comparative CT method and S18 recognizing both human and mouse transcripts were used for normalization.

### Statistical Analysis

Statistical analysis was performed with GraphPad Prism 7 software. Results were expressed as mean ± SD (*in vitro* studies) and mean ± SEM (*in vivo* studies). Statistical comparisons of the secretome at different passages were performed *via* one-way ANOVA with Bonferroni corrections test applied for multiple comparisons. *In vivo* studies and inhibition studies were analyzed by two-way ANOVA with Tukey corrections for multiple groups. p<0.05 was considered significant.

## Results

### Characterization of Contaminating Hematopoietic Cells in Bone Marrow-Derived MSC Culture

**Figure 1:**
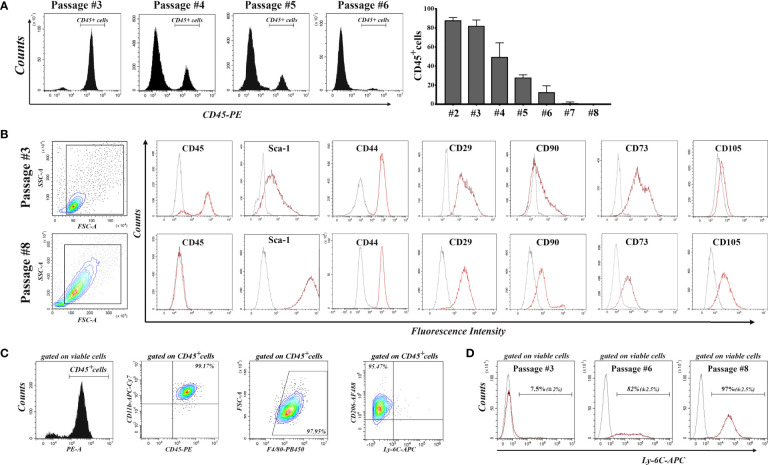
Flow-cytometry characterization of cells emerged from mouse bone marrow aspirate during serial passages until total depletion of CD45^pos^ cells. **(A)** The decrease in the percentage of CD45^pos^ cells with each passage. Note that the culture becomes completely depleted of CD45^pos^ cells after passage 6. Data are mean +/- S.D of at least 6 experiments. **(B)** Comparative analysis of the expression of cell markers in culture at passages 3 and 8. Note the presence of MSC markers (except CD105) on the cells at passage 3, when most of them are CD45^pos^ cells. At passage 8, the culture contained no CD45^pos^ cells, and cells are CD105^pos^. **(C)** Characterization of hematopoietic cells at passage 3. Note that all CD45^pos^ cells (around 80% of total viable cells in culture) are CD11b^pos^/F4-80^pos^/CD206^pos^/Ly-6C^neg^, being therefore asserted as anti-inflammatory cells. At least 3 different batches were analyzed and the results were similar. **(D)** Expression of Ly-6C in cell culture at increasing passages. Note that all MSC express Ly-6C in culture. Data are mean +/- S.D of at least 3 experiments.

Our strategy to purify mouse bone marrow-derived MSC in culture was based on serial passages through gentle trypsinization, by which MSC were detached and further propagated, while part of the hematopoietic cells remained attached to the substrate, being more resistant to trypsin. **Figure 1A** shows the decrease in the percentage of CD45^pos^ cells in bone marrow-derived cell culture with each passage, until the culture became negative to CD45, after passage 6. Within these first passages, a proliferation of hematopoietic cells was observed, as the percentage of CD45^pos^ cells increased from day 3 to day 5 after seeding (**Supplemental Figure 1**). We therefore named these cells bone marrow-derived proliferating hematopoietic cells (BM-PHC), a term that captures the origin and proliferative status of the contaminating CD45^pos^ cells in the mouse MSC culture at low passages. It is worth mentioning that a large variability in the time course of the culture purification was noted, with certain batches of serum producing hematopoietic-free cultures early than others (data not shown).

Comparative characterization of BM-PHC and MSC was done at passage #3 (at which BM-PHC represented more than 80% of total viable cells) and passage #8 (at which culture MSC was free of hematopoietic cells). The results showed that BM-PHC were smaller-sized, however they expressed the whole panel of markers that are usually used to characterize MSC, except the endoglin (CD105). Thus, both cell populations were positive for Sca-1, CD44, CD29, and CD73 and were CD90^low^ (**Figure 1B**). Comparative analysis of multipotency showed that BM-PHC could not generate adipocytes and chondrocytes *in vitro* (data not shown), as MSC did when cultured under appropriate conditions (28).

Further characterization of BM-PHC showed a population of CD11b^pos^/F4/80^pos^/CD206^pos^/Ly-6C^neg^ cells, which pointed towards an anti-inflammatory macrophage phenotype (**Figure 1C**). Importantly, Ly-6C and Ly-6G were not expressed on BM-PHC however, Ly-6C was noticed on MSC. As the percentage of CD45^pos^ cells decreased in culture, the percentage of Ly-6C^pos^ cells increased and all cells at passage #8 were positive to Ly-6C (**Figure 1D**). Therefore, Ly-6C is being proposed as a genuine marker for C57Bl/6 -derived MSC.

### Comparative Analysis of the Immunomodulatory Properties of BM-PHC and MSC

**Figure 2:**
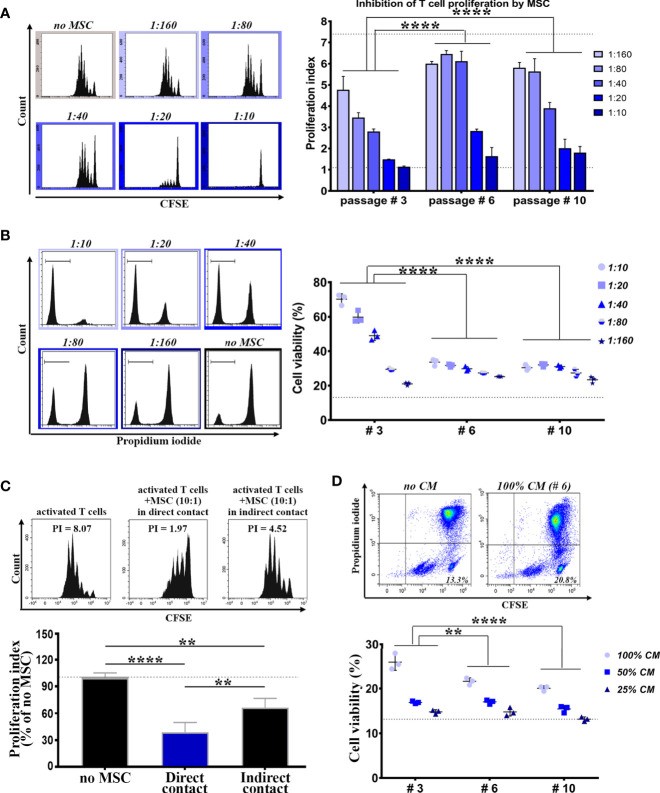
Comparative analysis of the immunomodulatory properties of cells within culture at low, intermediate and high passages. **(A)** The immunosuppressive effect of cells on splenic T cell proliferation in vitro. T cells were cultured in the presence of activating beads (1:1 ratio) and increasing numbers of irradiated cells, so that to span the interval of MSC: T cell ratio between 1:160 and 1:10 (constant number of T cells). Shown data represent mean +/- SD of one representative experiment performed in triplicates. At least three experiments were performed with similar results. **(B)** The pro-survival effect of cells on the viability of resting T cells *in vitro*. T cells were cultured in resting conditions in the presence of increasing numbers of irradiated cells. Shown data represent mean +/- SD of one representative experiment performed in triplicates. At least three experiments were performed with similar conclusions. **(C)** The suppressive effect of MSC (passage #6) on T cell proliferation *in vitro*, in the absence of cell-cell contact between MSC and T cells. **(D)** The pro-survival effect of the conditioned medium (CM) produced by cells on resting T cells *in vitro*. Shown data represent mean +/- SD of one representative experiment performed in triplicates. At least six experiments were performed with same conclusion. **p < 0.01, ****p < 0.001.

These small BM-PHC are the most abundant cells at passage #3, yet the function of these cells is unknown. Consequently, we comparatively evaluated the immunomodulatory properties of BM-PHC (as whole population at passage #3) and MSC at passages #6 (in which the percentage of contaminating cells was very low), and #10 (in which MSC had underwent several doublings in culture after total hematopoietic depletion), by co-culturing them with syngeneic splenic T cells in activating conditions. In corroboration to previous reports (36), our results showed a dose-dependent inhibitory effect of MSC on T cell proliferation (**Figure 2A** and **Supplemental Figure 2**), with no difference between MSC at passages #6 and #10. However, passage #3 was significantly more effective in suppressing T cell proliferation in comparison to passages #6 and #10, thus demonstrating a strong immunosuppressive effect of BM-PHC (**Figure 2A**).

We next evaluated the effects of BM-PHC and MSC on resting T cells in culture. The results showed a dose-dependent protective effect of MSC (**Figure 2B** and **Supplemental Figure 3**) at both passages #6 and #10, and again, a much more protective effect of BM-PHC (**Figure 2B**).

We then attempted to establish whether secreted soluble factors were involved in the immunosuppression properties of these cells. First, the impact of MSC on T cell proliferation was assessed at passage #6 in a transwell co-culture, without allowing direct cell contact. The results showed that, even in the absence of cell-to-cell contact, the immunosuppressive effect of MSC still remained significant (**Figure 2C**), thus emphasizing that the secretome produced by MSC was partially responsible for inhibition of T cell cycling. In addition, the secretome of either MSC or BM-PHC also had pro-survival effects on resting T cells in culture, albeit at lower extents than the cells (**Figure 2D**, in comparison to **Figure 2B**).

Together, these data demonstrated strong immunomodulatory properties of cells contaminating the MSC culture, which even surpassed the properties of MSC themselves, by promoting the viability of resting T cells and suppressing splenic T lymphocyte proliferation.

### Comparative Analysis of the Secretomes of MSC and BM-PHC

**Figure 3:**
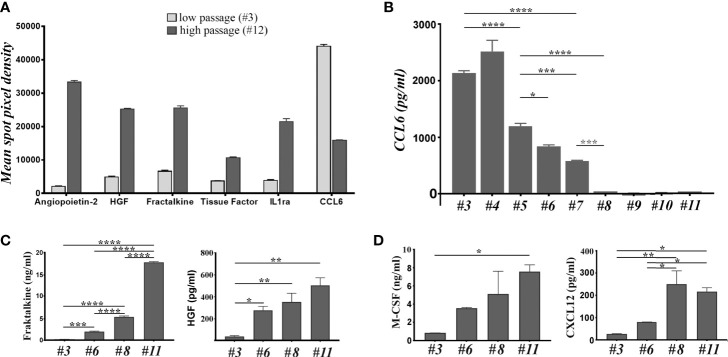
The analysis of soluble factors secreted by cells at low and high passages. ***(*A)** The relative expression level of 6 cytokines identified by cytokine array in the secretome of cells at 3 versus # 12. The depicted cytokines were selected from the 111 soluble proteins detected by cytokine array based on two considerations: a high level of expression and high differences between the two passages. **(B)** ELISA quantification of CCL-6 in the secretome of cells at increasing passages. Note the high level of CCL-6 at low passages and its loss after passage 7. The data represent the results of one representative experiment from 5 experiments with different batches and similar conclusions. **(C)** ELISA quantification of Fractalkine and HGF in the secretome of cells at different passages. The data represent the mean+/- S.D. @ of at least 3 experiments performed in duplicates. Note that Fractalkine is secreted by cell culture at all passages, while HGF is completely absent from the culture at low passage. **(D)** The LegendPlex quantification of M-CSF and CXCL12 in the secretome of cell culture at different passages. Data illustrates one experiment performed in duplicates. Two independent experiments with different batches were analyzed and the results were similar. *p < 0.05, **p < 0.01, ***p < 0.005, ****p < 0.001.

The above data showed that the immunomodulatory effects of MSC and BM-PHC could be reproduced to a certain extent by the soluble factors secreted by these cells. To search for candidate molecules involved in the immunomodulatory properties of these cells, their CM was assessed by cytokine array. Around 22 proteins were identified at high levels (**Supplemental Figure 4**), with 6 of them having considerable differences in the secretion level between the two cell types. Specifically, Angiopoietin-2 (Ang-2), Hepatocyte Growth Factor (HGF), Fractalkine, Tissue Factor and Interleukin-1 receptor antagonist (IL-1ra) were secreted at higher levels by MSC than BM-PHC. On contrary, CCL6 (a mouse C-C motif chemokine), with chemoattractant properties for macrophages, B and T lymphocytes and eosinophils (37), was secreted at higher level by BM-PHC (**Figure 3A**).

The different secretion level of these molecules was further assessed by ELISA, using four different batches at different passages. The results confirmed the gradual decrease of CCL6 level with increasing passage (**Figure 3B**), thus suggesting that BM-PHC was the source of CCL6. Quantification of Ang-2 level in various batches at different passages showed batch-dependent secretion patterns, with some batches secreting high levels of Ang-2 at high passages (**Supplemental Figure 4**), and other batches secreting very low levels at all passages (**Supplemental Figure 5**). Quantification of IL-1ra revealed a high, yet wide-ranging secretion level between passages, with no validated increased level in MSC as compared to BM-PHC (**Supplemental Table 1**). On contrary, the increase in the secreted levels of Fractalkine and HGF with increasing passage was validated by ELISA, and very low levels of molecules were detected in BM-PHC (**Figure 3C** and **Supplemental Figure 5**). Similarly, Tissue Factor increased in MSC with passages (**Supplemental Figure 5**); however, it is worth mentioning that the levels of Tissue Factor were very low, ranging from 5 - 50 pg/ml. This data is important for intravascular therapeutic delivery of MSC, as Tissue Factor is the major determinant of cell product hemocompatibility (38). On the other hand, both cell types secreted high levels of Prostaglandin E2 (PGE2) at all passages, which were 3 orders of magnitude above the Tissue Factor level, with a median of around 4 ng/ml (data not shown). PGE2 was reported to be markedly increased in the inflammatory settings and has roles in inhibition of cytotoxic T cell development, division and function (39).

To get deeper insights into the composition of the secretome, two bead-based multiplex assay panels were used to quantify several mouse cytokines specifically associated to T helper or myeloid stem cells. The results showed no or minimal secretion levels of IL-2, -4, -5, -6, -10, -13, -34, as well as of Interferon γ (IFN-γ), Tumor Necrosis Factor α (TNF-α), Tissue Growth Factor β1 (TGF-β1), Granulocyte/Macrophage Colony Stimulating Factor (GM-CSF) and Stem Cell Factor (SCF), which thus confirmed the data obtained by cytokine array (**Supplemental Table 1**). Instead, very high levels of Macrophage Colony Stimulating Factor (M-CSF) were found in the CM of both cell types, with significantly higher levels secreted by MSC as compared to BM-PHC (**Figure 3D**). Likewise, CXCL12/SDF-1, a chemokine involved in stem cell homing and T cell chemoattraction had been found in higher levels in MSC than in BM-PHC. Both M-CSF and CXCL12 have been previously reported as being secreted by MSC and involved in the control of survival and differentiation of bone marrow progenitor cells (40). A summary of all molecules identified in MSC secretome is illustrated in **Supplemental Table 1**.

### Candidate Molecules for the Immunosuppressive Effects of MSC and BM-PHC

**Figure 4:**
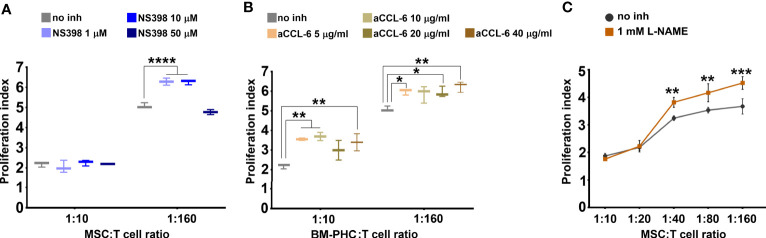
The effects of cells on T cell proliferation in the presence of inhibitors. **(A)** Effect of different doses of NS398, a specific COX-2 inhibitor, on reversing the inhibitory effect of MSC. Note the capacity of NS398 at 1 and 10 µM to partially reverse the MSC effect on T cell proliferation at the lowest MSC: T cell ratio. **(B)** Effect of different doses of CCL-6 neutralizing antibody on reversing the inhibitory effect of BM-PHC. Note the reversing effects of anti-CCL-6 at both BM-PHC: T cell ratios. The graphics in a-b illustrate a representative experiment from at least four experiments performed with different batches, with the same conclusions. **(C)** Effect of L-NAME, a specific NO synthase inhibitor, on reversing the inhibitory effect of MSC. The graphic illustrates a representative experiment from three experiments preformed with different L-NAME doses: 100 nM, 500 nM, and 1mM. No dose-dependent effect of L-NAME was observed, yet the three doses produced the same reversing effects. *p < 0.05, **p < 0.01, ***p < 0.005, ****p< 0.001.

The above data showed that BM-PHC secreted high levels of CCL6, whereas MSC secreted high levels of HGF, Fractalkine, M-CSF and CXCL12. However, both cells types secreted high levels of PGE2, previously reported to modulate the immunity (41) and the immunosuppressive properties of MSC (42). We therefore hypothesized that the immunosuppressive effect of both cell types is primarily mediated by PGE2, and to lesser extents by HGF and CCL6, which were differentially secreted by the two cell types. To test this hypothesis, proliferation studies of activated T cells in co-culture with MSC or BM-PHC in the presence of specific inhibitors of PGE2, HGF or CCL6 were done. BM-PHC were used for studying the effect of CCL6 and MSC for studying the effects of PGE2 and HGF. Our data showed that NS398, a specific COX-2 inhibitor, partially reversed the suppressive effect of MSC and increased the proliferation index of T cells at doses ranging from 1 to 10 µM (**Figure 4A**). This effect was apparent only at 1:160, and not 1:10, cell ratio (MSC: T cell), which thus suggested a partial contribution of PGE2 on the inhibitory effects of MSC on T cell proliferation *in vitro*.

Interestingly, inhibition of CCL6 by using a specific neutralizing antibody significantly attenuated the inhibitory effect of BM-PHC at both 1:160 and 1:10 cell ratios (**Figure 4B**). This data showed a major suppressive role of CCL6 on T cell proliferation *in vitro*, which has not been previously reported and might explain the enhanced immunomodulatory properties of BM-PHC over MSC. However, the specific blocking of CCR1 (reported as the putative receptor of CCL6) (37), using BX471 small molecule, totally suppressed T cell proliferation (**Supplemental Figure 6A**), which probably reflected the involvement of other CC chemokines, such as CCL3, or CCL5 (which are synthetized by T cells and also signalize through CCR1 (43, 44), in cell survival and proliferation.

Similarly, inhibition of HGF signaling using SGX523 (a specific c-Met inhibitor) negatively impacted the T cell proliferation *in vitro* (**Supplemental Figure 6B**), which pointed towards the important role of HGF in cell proliferation and survival. However, addition of recombinant HGF protein (50-100 ng/ml) on activated T cells did not produce inhibitory effects (data not shown), suggesting that HGF was not involved in the suppressive effect of MSC on T cell proliferation in culture.

Collectively, these data indicated that CCL6 secreted by BM-PHC, yet not by MSC, might explain the superior effects of BM-PHC in inhibiting activated T cell proliferation *in vitro*, as compared to MSC. PGE2, which was secreted at high levels by both cell types, was found to be partially involved in the inhibitory effects on T cell proliferation, yet the degree of inhibition did not point towards PGE2 as the major inhibitory molecule. We therefore assumed that T-cell suppression might be also mediated by factors induced in the presence of activated T cells, by the cross-talk between the two cell populations.

As nitric oxide (NO) was previously reported to inhibit T-cell proliferation *in vitro* (45, 46), we investigated the effects of L-NAME, a specific inhibitor of NO synthase. As shown in **Figure 4C**, 1 mM L-NAME partially reversed the immunosuppressive effects of MSC and its effect was more pronounced in the presence of low numbers of MSC (at MSC: T cell ratio of 1:40 and above). This data suggested that NO was also an important suppressive factor of T cells *in vitro*. However, complete recovery was not achieved, implying that a synergistic immunosuppressive mechanism of MSC on T cell proliferation did exist.

### Putative Mechanisms by Which MSC Induce Suppression of T Cell Proliferation

**Figure 5:**
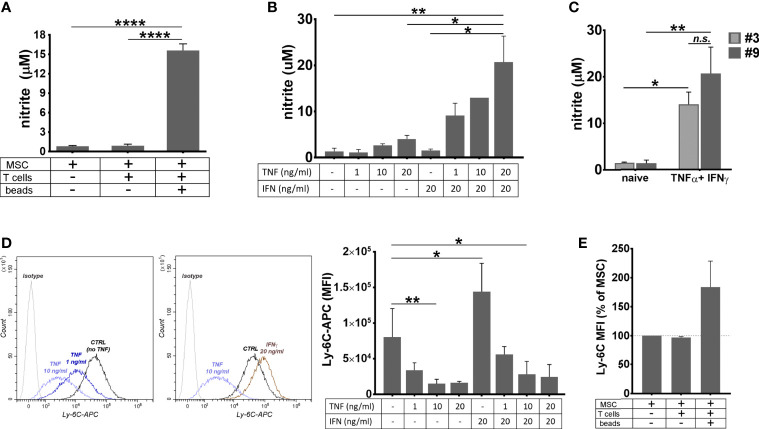
The behavior of MSC in pro-inflammatory conditions. **(A)** The level of nitrite secreted by MSC in basal conditions, and in the co-culture with resting or proliferating T cells. The values represent mean+/- S.D. @ of two independent experiments performed in triplicates. **(B)** The levels of nitrite secreted by MSC after 48 hours of culture in the presence of TNFα and IFNγ. Note the synergic effect of the two cytokines on the secreted NO level. The values represent mean +/S.D of four experiments performed in duplicates with different MSC batches at passages 7- 10. **(C)** The levels of nitrite secreted by cell culture at low and high passages. The values represent mean +/S.D of at least two experiments performed in duplicates. **(D)** The attenuation of Ly-6C expression on MSC after stimulation with TNF*α* in the presence or absence of IFN*γ*. Note that, while TNFα produced a dose-dependent decrease in the expression of Ly-6C in MSC culture, IFNγ had an inverse effect, however in the presence of both cytokines, MSC decrease Ly-6C expression. The values represent the mean +/- S.D. from the three independent experiments. Representative histograms showing Ly-6C expression in MSC with and without cytokines are also given. **(E)** Ly-6C expression on MSC in basal conditions, and in the co-culture with resting or proliferating T cells (n= 2 experiments). *p < 0.05, **p < 0.01, ****p < 0.001, n.s., not-significant.

Since NO is known as a highly unstable molecule, we next investigated the context in which NO secretion occurred in cultured MSC. To this aim, supernatants from naïve MSC culture, as well as from the 3-day co-culture of MSC with T cells in activating or quiescent conditions, were used for nitrite determination by Griess reaction. The results showed that MSC produced high levels of nitrite in the presence of activated T cells (**Figure 5A**). On contrary, neither naïve MSC, nor MSC in the presence of resting T cells did produce nitrite.

As MSC were previously reported to produce NO when activated by TNFα and IFNγ (46) and both these molecules were identified in the secretome of activated T cells (47), we measured the nitrite level in the culture medium of naïve MSC in the presence of various doses of TNFα and IFNγ, alone or in combination. The results showed small levels of NO secreted by MSC in the presence of TNFα, in a dose-dependent manner, and no NO secreted in the presence of IFNγ alone. However, the concomitant presence of the two cytokines in MSC culture resulted in a massive NO secretion (**Figure 5B**). No significant difference was found in the NO levels produced by MSC and BM-PHC (**Figure 5C**). It is therefore likely that, similar to PGE2, NO production was a common mechanism by which the two cell types induced the suppression of T- cell proliferation *in vitro*.

Given the anti-inflammatory behavior of MSC in the presence of inflammatory cytokines, we investigated whether the expression of pro-inflammatory protein Ly-6C was changed in MSC in our experimental setting. Flow-cytometry analysis revealed that TNFα produced a dose-dependent decrease in the expression of Ly-6C in MSC culture (**Figure 5D**). On contrary, IFNγ increased the Ly-6C expression in MSC culture, thus suggesting that the balance between the two cytokines dictates the overall expression of Ly-6C on MSC. We further determined Ly-6C expression in MSC in co-culture with activated T cells and found it increased, as compared to naïve MSC (**Figure 5E**). This data might be explained through increased level of INFγ being secreted over TNFα in this experimental setting.

By summarizing, the factors by which MSC exerted the immunosuppressive effects on T cell proliferation *in vitro* appear to involve the constitutive secretion of PGE2 and the induced secretion of NO. Besides these two molecules, BM-PHC appear to exert the immunosuppressive effects on T cell proliferation *in vitro* also by CCL6.

### Capacity of BM-PHC and MSC to Inhibit Xenogeneic Tumor Formation in Immunocompetent Mice

**Figure 6:**
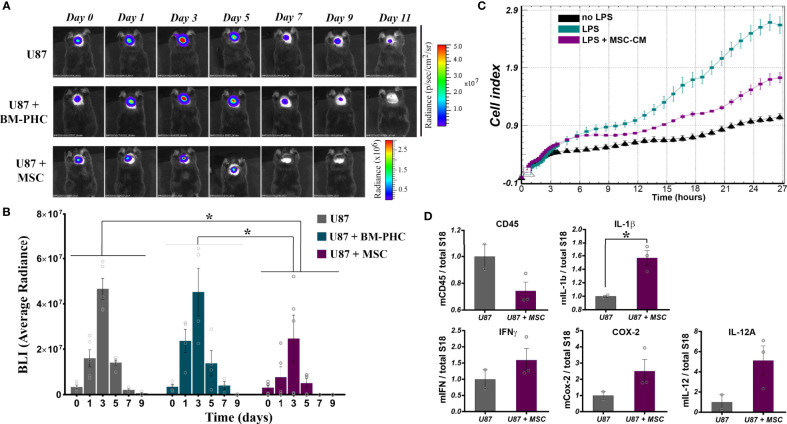
The effects of BM-PHC and MSC on tumor development in immunocompetent mice. **(A)** A representative bioluminescent image of C57Bl/6 mice subcutaneously transplanted with U87 cells alone (U87) or in the presence of BM-PHC or MSC is given above for each time point analyzed. **(B)** The diagram illustrates the bioluminescence signal of the tumor cells in all three groups, determined as average radiance. Values represent means+/-SEM of n=4-5 animals/group. **(C)** Dynamic assessment (original recording) of the effect of MSC secretome on macrophage activation in the presence of LPS. The recording represents mean values obtained from triplicates of one representative experiment from 2 experiments. **(D)** Quantification by real time RT-PCR (n = 2-3 per group) of the relative expression of genes associated with inflammation in cellular aggregates of tumor cells extracted after 5 days from implantation. *p < 0.05.

Having the strong immunosuppressive effects of BM-PHC and MSC on T cell proliferation *in vitro*, we attempted to evaluate whether these two cell types would induce tolerance to tumor development in a model of xenotransplantation of tumor cells in adult mice with intact immune system. To this aim, 2 x 10^6^ U-87 MG-luc2 cells (human glioblastoma - derived cells that constitutively expresses Luciferase) were subcutaneously injected in C57Bl/6J mice, either alone, or mixed with 1 x 10^6^ BM-PHC or MSC. *In vivo* bioluminescence imaging demonstrated that, in the absence of cell therapy, the tumor rejection occurred between days 3 and 9 in all groups, with complete rejection occurring by day 11 (**Figures 6A, B**). Similar results with U-87 MG-luc2 cells injected into immunocompetent mice were previously reported (48). The group receiving tumor cells mixed with BM-PHC showed a transient increase in the luminescent signal within the first three days after transplant, followed by tumor rejection by day 9, a pattern similar to control group. This data suggests no significant effects of BM-PHC on tumor development.

In contrast, co-injection of U-87 with MSC resulted in a significantly more rapid rejection of the xenograft, indicating a direct anti-tumoral effect of MSC (**Figure 6A**). As tumor growth was visibly affected from the first day after implantation (**Figure 6B**), a direct impact of MSC on the innate immune cells invading the tumor was assumed. In vitro investigation of the effect of MSC secretome on the activation of macrophages showed a significant anti-inflammatory effect of MSC, by slowing down the cell index, indicative of macrophage activation in the presence of LPS (**Figure 6C**). A similar anti-inflammatory effect of MSC was noted *in vivo*, in the model of tumor xenotransplantation described above, where quantitative RNA analysis of tumors removed at five days after injection revealed a tendency of decrease in the CD45 mRNA level in U87+ MSC group, as compared to U87 group (**Figure 6D**). The level of CD45 gene expression was positively correlated with the immunohistochemically-quantified cell marker in solid tumors, as previously documented (49). Still, RT-qPCR analysis identified increased transcription of several pro-inflammatory genes e.g., IL-1β, IFNγ, COX-2, IL-12A (**Figure 6D**). A possible explanation for these results is that although MSC retained the anti-inflammatory properties *in vivo*, by reducing the number of hematopoietic cells infiltrated the transplant area, they underwent activation in the presence of tumor cells and consequently become polarized towards the inhibitory functionality for tumor development. However, due to the low number of animals used in this study, the statistical significance of the data was not reached. Therefore, additional studies are warranted to confirm this mechanism by which MSC act to inhibit tumor initiation *in vivo*.

Together, these data show that mouse bone marrow aspirate generates in culture two populations of proliferating cells with immunomodulatory properties, MSC and BM-PHC, which are valuable for therapeutic purposes. While both cell types inhibit the proliferation of activated T cells and promote the survival of resting T cells *in vitro*, the *in vivo* effects are divergent: MSC exert an anti-tumor effect, whereas BM-PHC may induce transplantation tolerance. Therefore, these two cell populations should be considered for cell therapy depending on the context.

## Discussion

The major findings of this study are summarized as follows: (i) bone marrow-derived MSC express high levels of Ly-6C; (ii) BM-PHC, the hematopoietic cells contaminating the MSC culture at initial passages, have strong immunomodulatory properties on T cells *in vitro*, which were partially mediated by CCL6; (iii) PGE_2_ and NO, secreted by both MSC and BM-PHC, are common mediators of the suppression of T cell proliferation *in vitro*; (iv) MSC, yet not BM-PHC, exert anti-tumoral effects *in vivo*.

Increasing data on the immunomodulatory effects of MSC have shown that the mechanisms of action were largely paracrine-mediated (50). However, substantial batch-to-batch variation, as well as differences based on donor, tissue of origin, culture conditions and passage were observed (51). We showed here that MSC secrete constitutively high levels of IL-1ra, Fractalkine, PGE2, HGF. Among them, PGE2 was validated as being involved in the immunosuppressive effects of MSC on T cells *in vitro*. As IL-1ra and fractalkine were acknowledged as molecules with opposite roles in lymphocyte recruitment (52, 53), their concomitant production by MSC may not produce a major impact on the immunosuppressive function. On the other hand, HGF was proven not to affect T cell proliferation *in vitro*. Neither addition of recombinant HGF, nor inhibition of HGF receptor in our co-culture system did reverse the immunosuppressive effects of MSC. This may not be surprising, as HGF mostly exerts its role on dendritic cells (54–56). Still, HGF has many other reported functions, such as induction of angiogenesis, promotion of cell proliferation and migration, and inhibition of apoptosis (57), which are accomplished through its receptor, c-Met. T cells were showed to express c-Met, which was reportedly involved in immune system activation against cancer cells overexpressing HGF (54). Our inhibitory studies showed that inhibition of c-Met by SGX523 negatively affected T lymphocytes, thus emphasizing that HGF-c-Met signaling was crucial for normal cellular processes both in MSC and T cells.

Another mechanism of MSC-mediated immunosuppression involves NO secretion (45, 46, 58). Our study showed that co-culture of MSC with activated T cells in the presence of L-NAME partially reversed the MSC inhibitory effect. It is important to emphasize that this effect was elicited by IFNγ and TNFα, which were actively secreted by activated T cells (59, 60), and in their absence MSC did not produced NO (40, 46). Indeed, our *in vitro* studies confirmed that only MSC stimulated with both IFNγ and TNFα produced high levels of nitrite in the culture medium.

In addition to the contribution of PGE2 and NO in MSC-mediated immunosuppression of T cell proliferation, we showed here a decline in Ly-6C expression on MSC in the presence of TNFα and an increase in the presence of IFNγ, which suggested that the anti- or pro-inflammatory behavior of MSC was decided by the balance between these two cytokines in various settings (19). The presence of Ly-6C on MSC has not been acknowledged before. It would be interesting to find out if this expression is a particularity of these cells (C57Bl/6-derived MSC) or is a more generalized characteristic of mouse MSC.

We also report here that BM-PHC reveal similarities with anti-inflammatory macrophages and share many characteristics with MSC, in terms of surface markers and immunomodulatory properties *in vitro*. These CD45^pos^ cells with positive expression of F4/80 and negative expression of Ly-6C and Ly-6G are different from the cell subset termed myeloid-derived suppressor cells (MDSC), which are basically inflammatory cells, and have been defined based on the high expression levels of Ly-6C (monocyte-derived MDSC) and/or Ly-6G (polymorphonuclear-derived MDSC) and lack of F4/80 molecules (61–64). However, these cells share several biological properties with MDSC, such as myeloid origin, *in vitro* proliferation, and suppressive potential for T cell proliferation. Other similarities of BM-PHC with MDSC refer to the mechanisms they use to suppress immune functions, as previous reports indicated that MDSC used inducible NO synthase and arginase for suppressing immune functions (65). Besides, up-regulation of COX-2 and PGE2 by MDSC had also been mentioned among the mechanisms of immunosuppression (39).

BM-PHC secreted high levels of CCL6, which was partially involved in the suppressive effect of these cells on T cell proliferation. As CCL6 was not secreted by MSC, this molecule might explain the superior immunosuppressive effects of BM-PHC over MSC. In harmony with our results, a previous study suggested a role of CCL6 in the antileukemic immune response and CCL6 down-regulation as a mechanism adopted by leukemic cells to evade the immune system (66). Furthermore, another study reported an apoptotic effect of CCL6 on several cell lines (67).

In conclusion, multiple cell populations with immunomodulatory properties can be obtained from bone marrow aspirate. They all may be valuable for therapeutic purposes, however the individual effect of each of them should be established in disease-relevant contexts.

A possible debating conclusion based on results reported in this paper is the antitumor effect of MSC *in vivo*. Extensive studies have been previously conducted and conflicting results have been reported with regards to the role of MSC in cancer therapy (68). On the one hand, there are studies boosting the conceptualization of MSC-based experimental cancer therapy by showing that MSC prevented tumor progression and metastasis though inhibiting angiogenesis or suppressing immune responses (69–73). On the other hand, several other studies reported the pro-tumorigenic properties of MSC (74–77). Similarly, although in a different context, MSC were demonstrated to delay the allograft rejection and generate a local immune privileged site (78).

Another debate that may also hinder the therapeutic potential of MSC is the significant safety concerns regarding the possible long-term tumor growth after MSC infusion, as previously reported in mice (79). Such *in vivo* spontaneous malignant transformation of mouse MSC have been previously documented particularly after long-term *in vitro* culture (80, 81), which sustained the hypothesis that cell characteristics are dynamics and change depending on intracellular and extracellular stimuli. In our experimental setting, MSC co-administrated with tumor cells generated a more rapid xenograft rejection in immunocompetent mice. While MSC apparently decreased the murine CD45 expression inside the tumor, the tumor microenvironment induced MSC polarization towards the inhibitory functionality, which resulted in rapid tumor annihilation. However, in this paper we have only focused on the fate of tumor cells, yet not followed the long-term effects of MSC transplant, therefore we cannot deliberate on malignant transformation of MSC *in vivo*. However, MSC remain unquestionably a promising therapy option for a variety of diseases, yet despite numerous *in vitro* and *in vivo* studies, there is much more that is still unknown and as such, more research and observations will be necessary to investigate the long-term effects of MSC therapies.

## Data Availability Statement

The raw data supporting the conclusions of this article will be made available by the authors, without undue reservation.

## Ethics Statement

The animal study was reviewed and approved by the Ethical Committee of ICBP.

## Author Contributions

C-IM, MP, CN, ER, SP, and AB performed experiments. C-IM, MP, and AB analyzed and interpreted the data. AB designed the work and wrote the manuscript. All authors contributed to the article and approved the submitted version.

## Funding

This work was supported by a project co-financed by the European Regional Development Fund through the Competitiveness Operational Program 2014-2020 (POC-A.1-A.1.1.4-E-2015, ID: P 37 668, acronym DIABETER) and Romanian Ministry of Education (PN-III-P1-1.1-PD-2016-1903, contract no 133PD/2018 and PN-III-P4-ID-PCE-2020-1340-contract 122/2021).

## Conflict of Interest

The authors declare that the research was conducted in the absence of any commercial or financial relationships that could be construed as a potential conflict of interest.

## Publisher’s Note

All claims expressed in this article are solely those of the authors and do not necessarily represent those of their affiliated organizations, or those of the publisher, the editors and the reviewers. Any product that may be evaluated in this article, or claim that may be made by its manufacturer, is not guaranteed or endorsed by the publisher.

## Abbreviations

MSC, mesenchymal stromal cells; BM-PHC, bone marrow-derived proliferating hematopoietic cells.
