# Supplementary material for: Identification of a Hematopoietic Cell Population Emerging From Mouse Bone Marrow With Proliferative Potential In Vitro and Immunomodulatory Capacity
Source: Front Immunol. 2021 Aug 3;12:698070. doi: 10.3389/fimmu.2021.698070 (PMC8368722; doi:10.3389/fimmu.2021.698070)
Supplement: Supplementary file 1 [file DataSheet_1.docx]

**­­­­Supplemental Material**

**
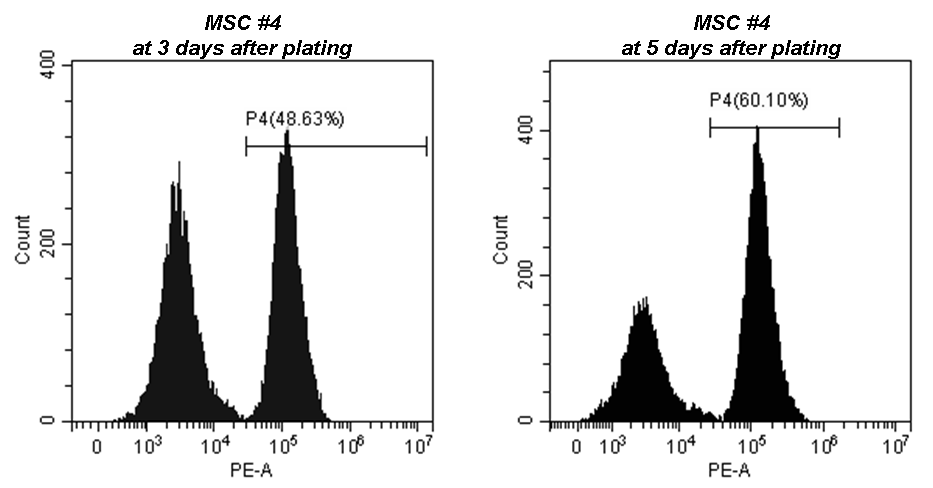
**

Supplementary Figure 1. The proliferation of hematopoietic cells between day 3 and day 5 after plating.


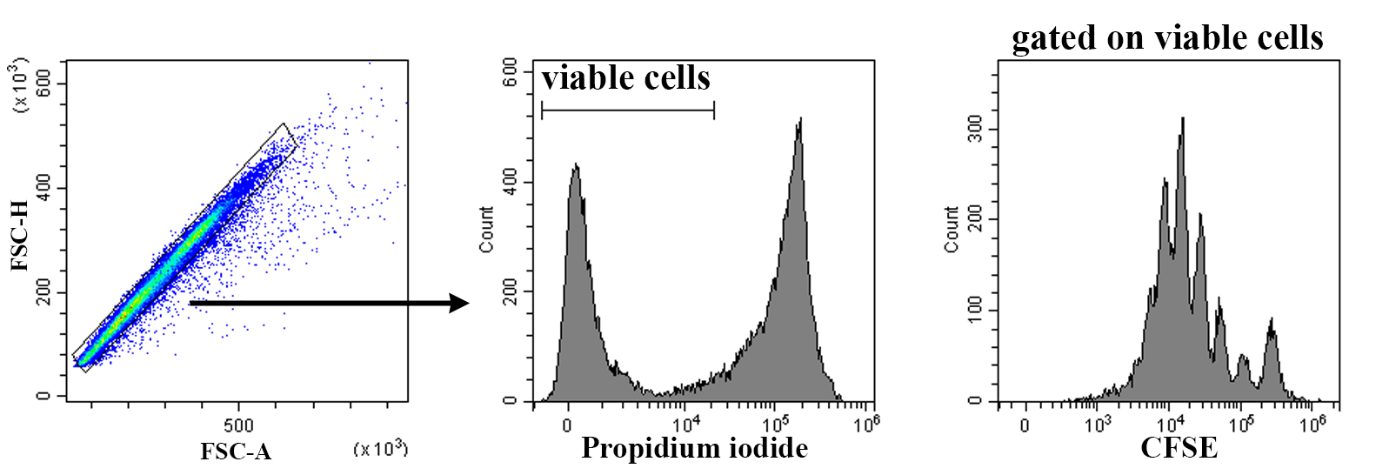


Supplementary Figure 2. Gate setting for the identification of CFSE-labeled of viable T cells.


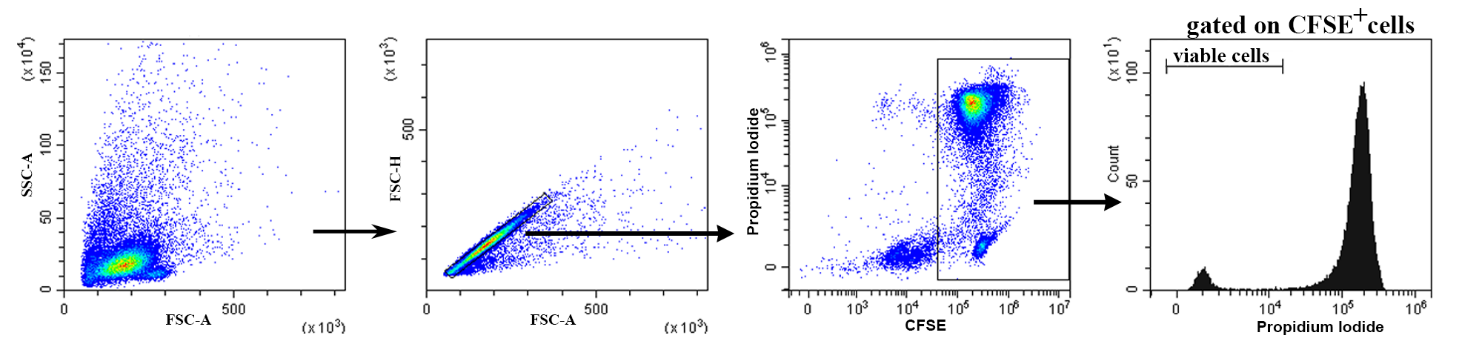


Supplementary Figure 3. Gate setting for the identification of viable T cells.


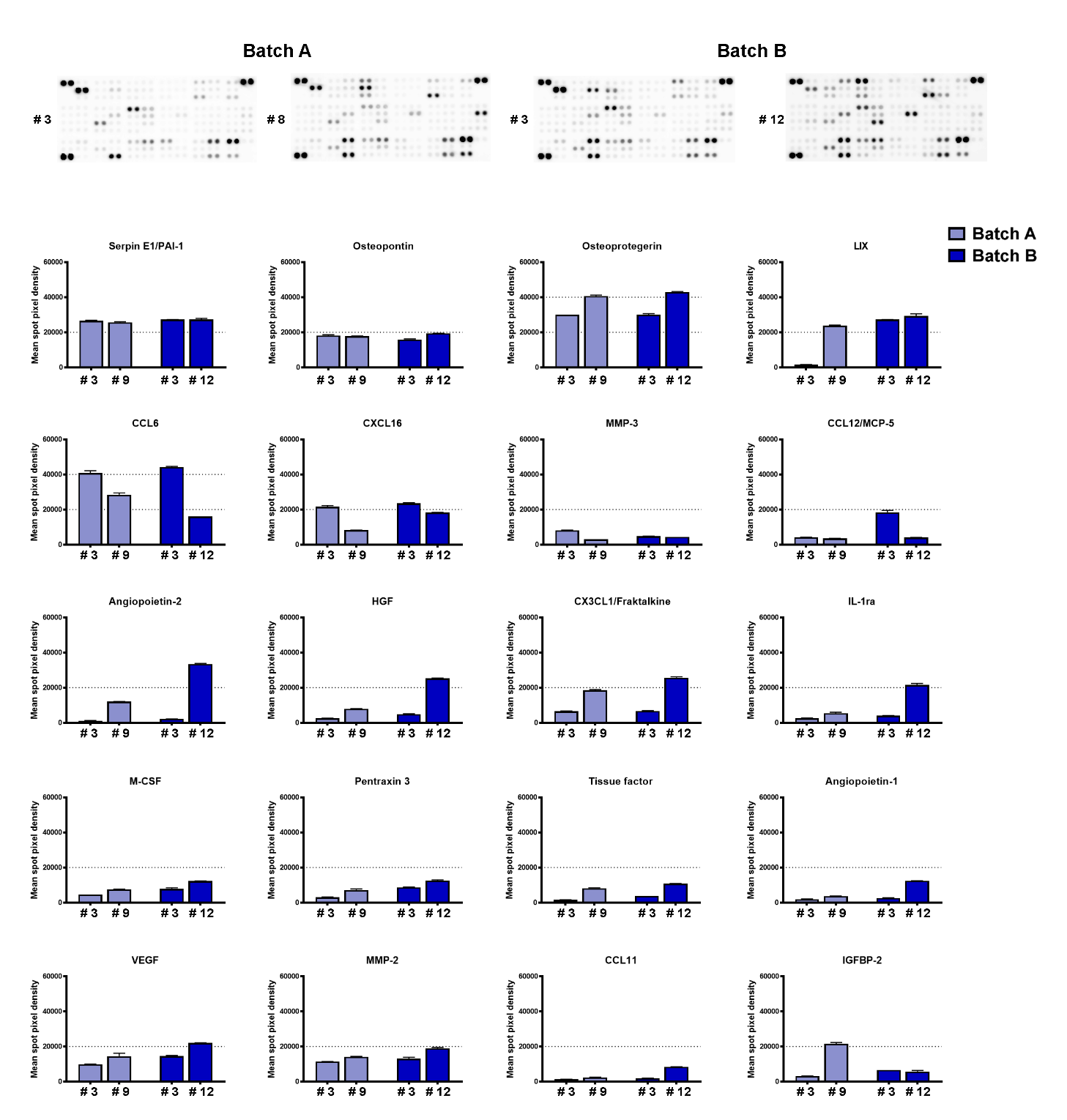


Supplementary Figure 4. The relative expression level of cytokines identified by cytokine array in the secretome cells produced by two batches at two passages.


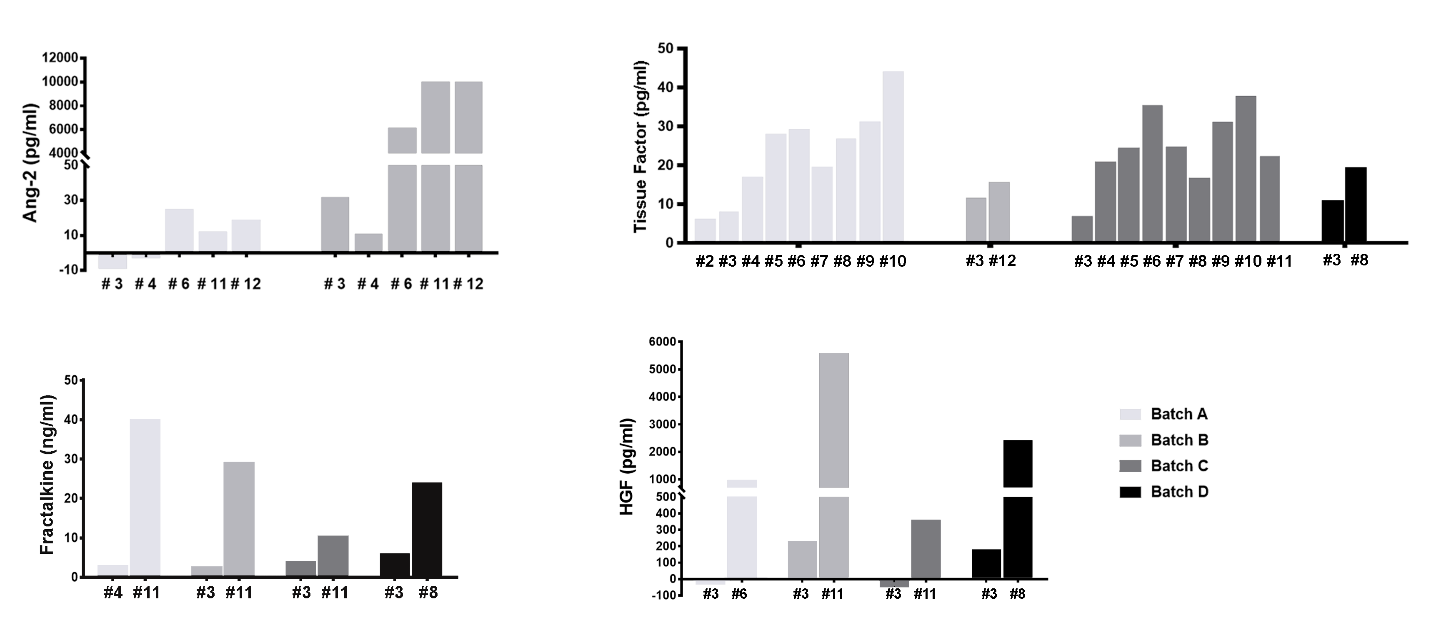


Supplementary Figure 5. ELISA quantification of molecule in the secretome produced by various batches of MSC at different passages.


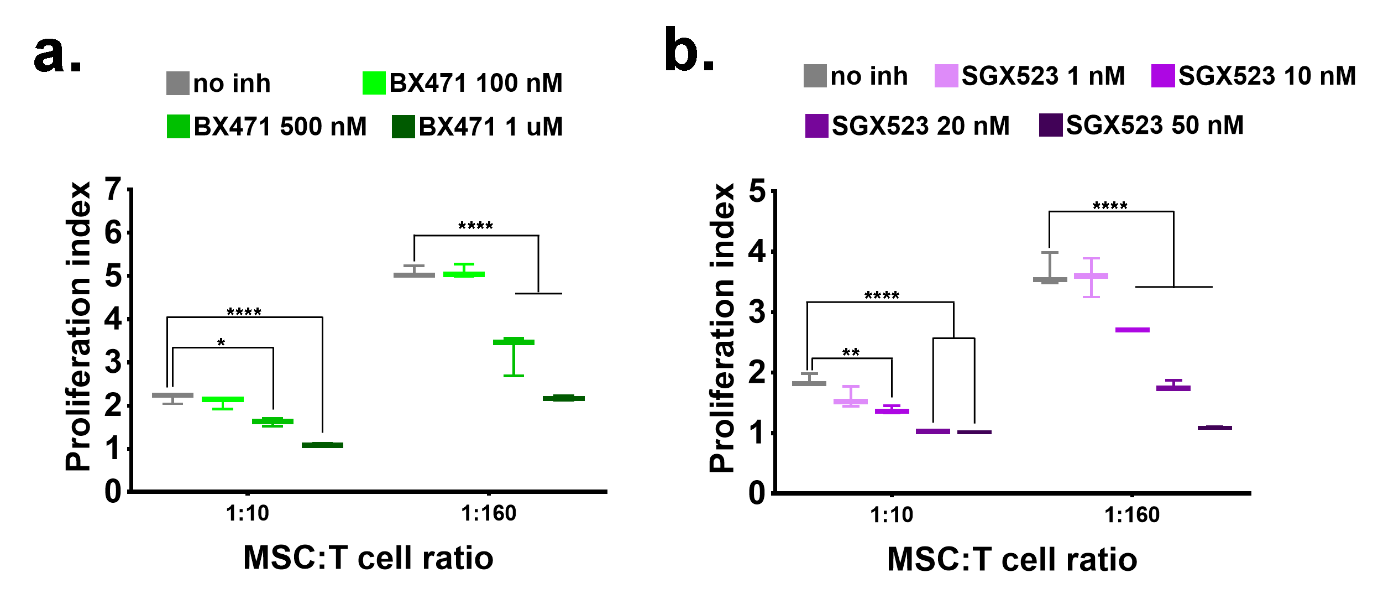


Supplementary Figure 6. The effects of MSC on T cell proliferation in the presence of (a) BX471 and (b) SGX 523.

| **Molecule** | **Secreted levels** | **Assay** | **Range** | **Observations** |
| --- | --- | --- | --- | --- |
| PGE2 | +++ | ELISA | up to 10 ng/ml | Fluctuating levels |
| IL1ra | +++ | ELISA | up to 3 ng/ml | Fluctuating levels |
| Fractalkine | +++ | ELISA | up to 40 ng/ml | Increased level with passage |
| Angiopoietin-2 | +++ | ELISA | up to 10 ng/ml | Batch dependent |
| HGF | +++ | ELISA | up to 5 ng/ml | Increased level with passage |
| CCL6 | +++ | ELISA | up to 3 ng/ml | Decreased level with passage |
| Tissue Factor | + | ELISA | up to 50 pg/ml | Increased level with passage |
| M-CSF | +++ | LegendPlex | up to 8 ng/ml | Increased level with passage |
| CXCL12 | ++ | LegendPlex | up to 300 pg/ml | Increased level with passage |
| IL-34 | + | LegendPlex | up to 50 pg/ml | Increased level with passage |
| IL-6 | + | LegendPlex, Array | up to 50 pg/ml | Increased level with passage |
| TNFα | - | LegendPlex, Array |  |  |
| IL-5 | - | LegendPlex |  |  |
| GM-CSF | - | LegendPlex |  |  |
| TGF-$\beta$1 | - | LegendPlex |  |  |
| IFNg1 | - | LegendPlex |  |  |
| IL-2 | - | LegendPlex, Array |  |  |
| Il-4 | - | LegendPlex, Array |  |  |
| IL-10 | - | LegendPlex, Array |  |  |
| IL-13 | - | LegendPlex, Array |  |  |
| Serpin E1 | +++ | array |  |  |
| Osteopontin | +++ | array |  |  |
| Osteoprotegerin | +++ | array |  |  |
| LIX | +++ | array |  |  |
| CXCL16 | ++ | array |  |  |
| MMP-2 | ++ | array |  |  |
| VEGF | ++ | array |  |  |
| MMP-3 | + | array |  |  |
| CCL11 | + | array |  |  |
| IGPB-2 | + | array |  |  |
| Pentraxin-3 | + | array |  |  |
| IGFBP-6 | + | array |  |  |
| CCL12/MCP-5 | + | array |  | Batch dependent |
| MCP-1 | + | array |  | Batch dependent |
| Angiopoietin-1 | + | array |  | Batch dependent |

Supplemental Table 1. Molecules identified in MSC secretome.
